# Recent development of AAV-based gene therapies for inner ear disorders

**DOI:** 10.1038/s41434-020-0155-7

**Published:** 2020-05-18

**Authors:** Yiyang Lan, Yong Tao, Yunfeng Wang, Junzi Ke, Qiuxiang Yang, Xiaoyi Liu, Bing Su, Yiling Wu, Chao-Po Lin, Guisheng Zhong

**Affiliations:** 1 iHuman Institute, ShanghaiTech University, Shanghai, 201210 China; 2 School of Life Science and Technology, ShanghaiTech University, Shanghai, 201210 China; 3 Department of Otolaryngology-Head and Neck Surgery, Shanghai Ninth People’s Hospital, Shanghai Jiaotong University School of Medicine, Shanghai, 200011 China; 4 ENT institute and Otorhinolaryngology Department of Eye & ENT Hospital, NHC Key Laboratory of Hearing Medicine, Fudan University, Shanghai, 200031 China

**Keywords:** Targeted gene repair, DNA damage and repair

## Abstract

Gene therapy for auditory diseases is gradually maturing. Recent progress in gene therapy treatments for genetic and acquired hearing loss has demonstrated the feasibility in animal models. However, a number of hurdles, such as lack of safe viral vector with high efficiency and specificity, robust deafness large animal models, translating animal studies to clinic etc., still remain to be solved. It is necessary to overcome these challenges in order to effectively recover auditory function in human patients. Here, we review the progress made in our group, especially our efforts to make more effective and cell type-specific viral vectors for targeting cochlea cells.

## Introduction

Hearing loss is a common neurological disorder. Both genetic causes and environmental factors, such as ototoxic chemicals, chronic ear infections, large noise, and aging, can lead to hearing loss and deafness. In China, around 2 of every 1000 children are born with a clinically significant hearing loss in one or both ears [1] and about 60,000 babies born in China each year have the hearing loss syndrome and about half of them has a genetic etiology.

Current therapies for hearing loss include hearing aids, middle ear prostheses/active implants to amplify the sound signal, or cochlear implants to directly stimulate spiral neurons [2]. These approaches enable patients to hear the outside sound to some degree, but the therapeutic result remains far from effective in restoring natural hearing, especially in case that patients have deficiencies in frequency sensitivity, natural sound perception, and speech discrimination in noisy environments [2]. Therefore, more effective methods are still urgently required for treating hearing loss.

The inner ear system contains three major types of functional cells: hair cells (HCs), supporting cells (SCs), and spiral ganglion neurons (SGNs), all of which play an important role in the process of hearing production and perception. There are two types of sensory HCs in the cochlea: the outer HCs (OHCs) and the inner HCs (IHCs). OHCs amplify sound signals, while IHCs convert the mechanical information carried by sound waves into electrical signals that are transmitted to the neurons [3].

## Genes required for cochlea function and hearing in cochlea cells

Many genes in both HCs and SCs play essential roles in the development or maintenance of cochlea and thus are involved in regulating the cochlea function and hearing. Here we provide a very brief information in this area. For more details, please refer other elegant review articles [4–6].

Transmembrane channel-like 1 (TMC1) is a pore-forming component of channels that participate in mechanoelectrical transduction of sound in cochlear and vestibular HCs [7]. Otoferlin (OTOF) is an essential protein in HC ribbon synapses [8], and was previously found playing an important role in regulating the mode of exocytosis in IHCs [9]. Mutations on these genes as well as others, like MYO7A, PCDH15, and POU4F3, cause the dysfunction of cochlea and lead to deafness [10–14].

Besides hair-cell gene mutations, SC gene mutations have also been linked to deafness. SCs are located at the bottom of the inner and OHCs, anchoring the sensory epithelium to the basilar membrane, thus playing a mechanical role in protecting and maintaining the surrounding environment for HCs. Some key deafness genes mainly express and have functions in SCs, such as GJB2, which affects the SC’s gap junction and is the most common hereditary deafness gene [1, 6, 15, 16]. In mammals, hair-cell loss due to environmental and genetic stress is thought to be permanent [17]. However, recent studies suggest that SCs are potential inner ear progenitor cells from which HCs can be regenerated [18, 19]. Therefore, SCs are a potential target for gene therapy, not only to correct inherited hearing defects, but also for hair-cell regeneration.

SGNs, located in a bony channel (Rosenthal’s canal) that spirals around axis of the cochlea (modiolus), are primary neurons of the auditory system. The HCs release glutamate neurotransmitters upon sound stimulation to bind NMDAR2 and mGluRIs on the SGN membrane to produce excitatory electricals. SGNs transmit these electrical signals to the auditory cortex through the eighth cranial nerve, enabling us to hear outside sound. Thus, SGNs, as the bridge between HCs and brain, are required for normal hearing. Unfortunately, noise exposure, ototoxic drugs, and genetic factors can cause the irreversible SGN damage or death, and thus the communication between HCs and brain is disturbed, leading to sensorineural hearing loss [17].

## A brief gene therapy history in auditory disease

In recent years, gene therapy has emerged as an important method to treat inherited diseases (Table 1). Although 140 deafness-associated alleles have been identified, few treatments are available to slow or reverse genetic deafness. Clearly, there is an urgent need to develop biotherapies for restoring auditory function. Among them, the gene therapy has become the most promising therapy for hereditary deafness [5]. The inner ear is an ideal target for gene therapy, and many viral and nonviral vectors have been developed for the transmission of genetic material in the cochlea [20]. The adeno-associated virus (AAV) is widely used in gene therapy due to its high infection efficiency, low pathogenicity and toxicity, sustained expression of the carried genes, as well as its simple, cheap, and fast production [21–24]. Several studies have achieved good results by AAV vector-mediated gene therapy using animal models with different mutated genes in several types of cells in cochlea [2, 21, 25–30].

**Table 1:** The brief history of gene therapy in hearing diseases.

Animal model	Treatment reagent	Injection time and delivery method	Ave. ABR improvement (best freq.) and treatment efficacy	Targeted cells and major morphological improvement
Vglut3^−/−^ mice [2]	AAV1-Vglut3	P1–3 and P10 Route: AC and RWM	~50 dB (90 dB of control) Lasted for 3–6 months	IHCs/Improve the morphology of partial afferent IHC ribbon synapses.
Kcnq1^−/−^ mice [54]	AAV1-Kcnq1	P0–2 Route: Scala media	~45 dB (90 dB of control) Lasted for 4–6 months	SV marginal cells/rescue the collapse of Reissner’s membrane death of HCs and cells in the SG.
*MsrB3*^*−/−*^ mice [55]	AAV2/1-MsrB3	E12.5 Route: in utero	~40–50 dB	IHCs and OHCs/recovery hearing and the morphology of the stereociliary bundles.
Slc26a4^−/−^ and Slc26a4^tm1Dontuh/ tm1Dontuh^ mice [56]	rAAV2/1-Slc26a4	E12.5 Route: in utero	~20–40 dB(8–12 weeks)	IHCs, OHCs and stria vascularis/restored hearing phenotypes included normal hearing and progressive hearing loss.
Gjb2cKO mice Cx26^fl/fl^P0-Cre [57]	AAV5-Cx26	P0 and P42 Route: RWM	~20 dB in P0 (100 dB of control mice) ~0 dB in P42	IHC, OHCs, and SCs/rescue the formation of organ of Corti and HCs. No morphology change in P42.
Whrn^wi/wi^ mice [58]	AAV2/8-whirilin	P1–5 Route: PSC	~20 dB Lasted for 4 months Rescue the vestibular function	IHCs/rescue the morphology and function of stereociliary bundles and temporarily the death of IHCs.
*Clrn1*^*ex4/–*^ *mice* [28]	AAV2/8-*Clrn1*	P1–P3 Route: RWM	~30–40 dB	IHCs and OHCs/restored hearing phenotypes included normal hearing as well as the synaptic ribbons.
*Clrn1*^*–/–*^*-TgAC1 mice* [59]	AAV2/8-*Clrn1*	P1–P3 Route: RWM	~30–40 dB Lasted for 5 months	IHCs and OHCs/restored the hair bundle structure and hearing.
Usher1c (c.216G>A) [29]	AAV2-harmonin	P0–1 and P10–12 Route: RWM	~50–60 dB (110 dB of control) Lasted for 6 months	IHCs and OHCs/rescue the function of stereociliary bundles and death of HCs.
Otof^−/−^ mice [21]	Dual AAV	P10 and P17 and P30 Route: RWM	~30–40 dB Lasted for 5–6 months	IHCs/restore the number of ribbons by promoting their production.
Otof^−/−^ mice [60]	Dual AAV2/6half-vector	P6–7 Route: RWM	~50–60 dB(110 dB of control)	IHCs/restore the exocytosis function of IHC and partially the number of ribbons.
TMC^−/−^ mice [61]	AAV2/1-Cba-Tmc	P0–2 Route: RWM	~20–30 dB (110 dB of control)	IHCs and OHCs/restore the sensory transduction current of HCs, SCs, and SGs.
TMC^−/−^ mice [62]	sAAV-Tmc1	P0–2 Route: RWM	~50–60 dB (110 dB of control) Lasted for 3 months Rescue the vestibular function	IHCs and OHCs/rescue the function of stereociliary bundles, sensory transduction current, and death of HCs.
Tmc1^Bth/+^ mice [63]	Cas9: gRNA	P1 Route:Scala media	~20–30 dB	IHCs and OHCs/rescue the death of HCs.
Tmc1^Bth/+^ mice [31]	rAAV2/9miTmc1	P0–2 Route: RWM	~30–40 dB	IHCs and OHCs/rescue the number of IHCs and OHCs partially.
Tmc1^Bth/+^ mice [27]	AAV-SaCas9-KKH	P1 Route: Scala media	~30–40 dB	IHCs and OHCs/rescue the morphology of stereociliary bundles and the death of IHCs.
Tmc1^Bth/+^ mice3	AAV9.miTmc1	P15–16 and P56–60 and P84–90 Route: RWM + SF	~30–40 dB No difference in P84–90	IHCs, OHCs, and SV/rescue the morphology of stereociliary bundles and temporarily the death of IHCs.

*PSC* posterior semicircular canal, *AC* apical cochleostomy, *SF* semicircular fenestration.

Akil et al. loaded the VGLUT3 gene with AAV1 vectors to VGLUT3 knockout neonatal mice that displayed deafness by round window membrane (RWM) injection [2]. VGLUT3 gene was strongly expressed in whole cochlea. In addition, acoustic brainstem response (ABR) experiments showed that VGLUT3 overexpression with AAV vector successfully rescued the hearing phenotype in VGLUT3 knockout mice. Murine Beethoven (Bth) mutation (Tmc1 c.1235T>A [p.Met412Lys]) leads to the autosomal-dominant hearing loss. Shibata et al. used rAAV2/9 as the viral vector to deliver designed artificial microRNAs to rescue the progressive hearing loss [31]. In their study, rAAV2/9 predominantly localized to IHCs with about 74% efficiency of infection and the hearing function get some recovery as tested by ABR and distortion product otoacoustic emissions. Notably, many conventional AAV serotypes can transduce IHCs with high efficiency, but still exhibit no or very low transducing efficiency in OHCs. In 2017, a breakthrough study carried by Landegger et al.’s group demonstrated that Anc80L65 transduced both IHCs and OHCs in mice with very high efficiency, a substantial improvement over conventional AAV vectors [32]. Anc80L65 successfully delivered wild-type Ush1c into the inner ears of the neonatal Ush1c c.216G>A mice model [29]. Taking the advantage of Anc80L65 transducing HCs, Pan et al. showed the most complete recovery of auditory and vestibular function with gene therapy approach with AAVs [29].

## The challenges in the cochlea gene therapy

Despite of many exciting advances in gene therapy for deafness in animal models, there is still a long way to go before it can be applied to deafness in humans. There are currently more than 20 clinical trials for hearing loss therapies in the United States with six potential therapeutic molecules. Intriguingly, there is one clinical trial involving gene therapy for auditory diseases [33]. Moreover, a number of AAV-related gene therapy drugs have been approved by the U.S. FDA, which fully proves the clinical potential of AAV. In the field of hearing, however, there is no clinical drug based on AAV. As mentioned above, there is some success in gene therapy animal studies with AAV delivery. However, the specificity and efficiency of these viruses remain weak and may cause unwanted side effects by expressing genes in other untargeted cells.

It is feasible to systematically characterize the specificity of different AAVs to transduce the different cell types in the cochlea. An elegant study showed that AAVs specifically transduced different types of retina in both mice and nonhuman primate under the control of different gene modulator components [34]. In China, Li et al.’s group at Fudan University screened the available AAV variants to target the SCs in cochlea, which are essential for the function of both HCs and SGNs and have the potential to transdifferentiate to hair-cell-like cells. They found that AAV9-PHP.eB showed relatively high transduction efficacy in both OHCs and IHCs, and this is consistent with the results from a recent study performed at Lee et al.’s group [35–37]. They found that AAV-DJ had relatively high efficiency in SCs, surpassing what has been reported previously [35, 38]. However, the existing AAV variants do not transduce the cochlea cells in an efficient way and especially SCs are not sufficiently targeted by these AAVs [37–39]. Thus, in order to make the gene therapy with AAVs, it is necessary to generate new AAV variants which should have two properties, high transducing efficiency and specificity.

## The development of AAV-ie

To achieve this, we employed a strategy similar to a previous study [22] and aim to discover AAV variants with high transducing efficiency by inserting select peptides into an AAV vector and tested the transducing efficiency in the in vitro cell culture and in vivo animals [40]. AAV vectors can be successfully delivered to the inner ear to transduce cochlea cells by injection through RWM [41]. Thus, the AAV needs to cross a mesothelial cell layer to infect HCs and SCs. We reasoned that novel AAV variants with the ability to cross the mesothelial cell layer may increase gene transfer efficiency [40]. Since an earlier study demonstrated that the insertion of a peptide (DGTLAVPFK) helped the new AAV vector cross the blood–brain barrier [22], we inserted the DGTLAVPFK peptide into the VP1 capsid of AAV-DJ and found that the new AAV variant, which they named as AAV-ie (inner ear), dramatically increase the transducing rate to 80% of SCs in cochlea [40].

**Fig. 1:**
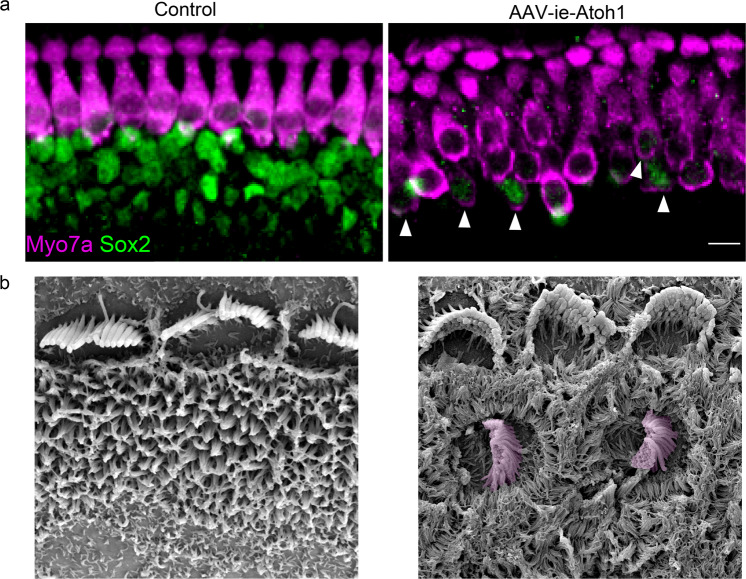
Adeno-associated virus-inner ear-*Atoh1* (AAV-ie-*Atoh1*) induces new hair cells (HCs) in vivo with stereocilia. **a** Representative confocal projection image of control and AAV-ie-*Atoh1* cochlea. Scale bar, 10 µm. **b** Scanning electron microscopy (SEM) images of AAV-ie- and AAV-ie-*Atoh1-*injected cochlea at apical regions. Regenerated HC-like cells were artificially colored magenta.

Cochlear SCs contain different cell types: Hensen’s cells, Deiters cells, pillar cells, inner phalangeal cells, and inner border cells. We found that high-dose AAV-ie infected all cell types of SCs with high efficiency without obvious toxicity to the cochlea function and auditory behaviors. Manipulation of signaling pathways and transcription factors such as gene *Atoh1* can lead to transdifferentiation of SC into HCs [42]. To assess the potential of the AAV-ie vector for HC regeneration, we used AAV-ie-*Atoh1*-NLS-mNeonGreen (AAV-ie-*Atoh1*) to deliver mouse *Atoh1* into the cochlea. New hair-cell-like cells were generated in the AAV-ie-*Atoh1* group as unambiguously demonstrated by the immunofluorescence labeling and SEM experiments (Fig. 1). The HC regeneration by the Atoh1 overexpression with AAV-ie is comparable with a previous genetic study that used *Foxg1*-Cre-mediated *Atoh1* overexpression mice, indicating AAV-ie is a powerful tool to deliver genes into SCs and could represent a potential tool to be used as HC regeneration. Indeed, we further demonstrated the newly generated hair-cell-like cells displayed excitable membrane properties relatively similar to the electrophysiological properties of HCs [40]. Using ex vivo human samples taken from ear surgery, we further demonstrated that AAV-ie can transduce the SCs in human utricle SCs. Recent collaborative experiments show that AAV-ie can transdifferentiate human utricle SCs to hair-cell-like cells in in vitro culture (data not shown). To our knowledge, this is the first study to use AAV as the deliver tool to show the unambiguous hair-cell-like cell regeneration in both rodent animal cochlea and culture human utricle cultures. Thus, AAV-ie may hold the potential for correcting genetic hearing impairment of SCs and also for HC regeneration to treat environmental and age-induced hearing loss or genetic auditory diseases given that in general AAVs have the lowest toxicity as viral vectors.

We reported that AAV-ie not only transduced SCs but also HCs in both animal models and human utricle samples. The nonspecific transducing properties of AAV-ie may limit it as an appropriate vector to deliver genes to SCs to treat either genetic or acquired hearing loss. Thus, further optimization of AAV variants to increase the transducing efficiency and specificity as gene transfer vectors for clinical use is much needed. We will discuss our current efforts to achieve the above goal.

## Improving the AAV efficiency

**Fig. 2:**
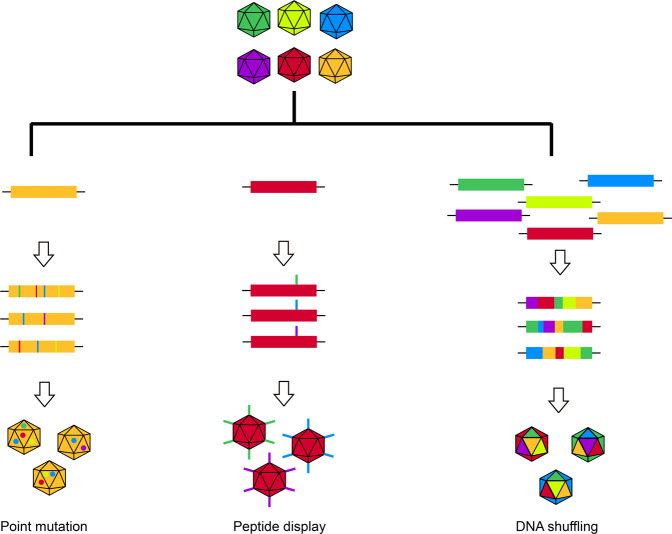
The strategies used to change the capsid to increase the transducing efficiency of AAV variants.

The existing AAV variants did not evolve for the purposes of highly transduce the cochlea cells, especially SCs [35, 37, 39]. Modification of these AAV variants to improve their efficacy and specificity of their potential use in inner ear gene therapy is much needed. There are many strategies to increase the transducing efficiency of AAV variants as illustrated in Fig. 2. Rational design of point mutations may increase the chance of AAV variants trafficking to the nucleus by the lack of AAV capsid ubiquitination [43–46]. Another strategy is to randomly fragment and reassemble the capsid genome of wild-type AAV serotypes 1–13 by PCR to generate a chimeric capsid library. Newly generated capsids may give the synthetic AAV different properties, such as tissue tropism and transducing efficiency [47–50]. In addition to the above two methods, peptides can be inserted into specific regions of AAV capsids to change their properties and several AAV variants are found to be highly efficient to transduce cells in central nervous system and in cochlea [22, 23, 40]. These efforts to optimize the capsids have led to the development of new AAV variants that are capable of high efficiency transduction at lower doses, and this increases the chance of their use in human gene therapy.

## Achieving the AAV specificity

AAV variants displaying high transducing efficiency often lack specificity and may bring severe side effects. To circumvent this shortcoming, efforts are needed to make the expression of interested genes in specific types of cells. The production of cell-targeted AAV can be achieved by selecting cell-specific promoters [22]. To achieve the specificity of AAV variants, we searched the literature for genes specifically expressed in the different types of cochlea cells, HCs, SCs, and SGNs (Table 2). Our single cell sequencing data are consistent with this information (data not shown).

**Table 2:** Genes are specifically expressed in cochlea cells.

HCs	SCs	SGNs
Ocm [64]	Gjb2 [30]	Syn [65]
Slc26a5 [66]	Lgr5 [67, 68]	NeuN [69]
Otof [70]	GFAP [71]	Map2 [72]
Atp2a3 [70, 73]	Fgfr3 [74]	Tuj1 [75]
Tpbgl [70]	Tak1 [76]	Calb1 [77]
Dnajc5b [70]	Sox21 [78]	Calb2 [79]
Myo7a [80]	Sox2 [81, 82]	Nos1 [83]
Myo6 [84]	PLP1 [68]	Runx1
Zfp [85]	CD44 [86]	Prph [87]
Tmc1 [73, 88]	Prox1 [89, 90]	Cacna1h [91]
Tmc2 [88]	CX30 [92]	Slc6a4 [83]
Cabp2 [93]	Aquaporin4 [94]	Grm8 [95]
Brip1 [96]		Brn3a [97]
Zmat3 [98]		Trim54
Strip2 [96]		

**Fig. 3:**
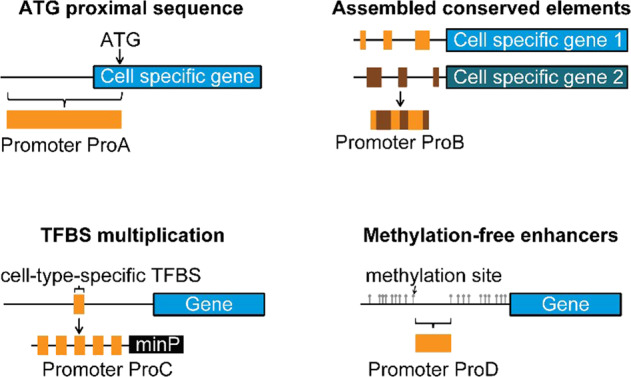
Different strategies are used in constructing the synthetic promoter. ProA: A 5′ end of a specific type of inner ear cell-specific expressed gene −3000 to 500 bp. ATG is the translation start site. ProB: A phylogenetic conserved sequence before the transcription initiation site of a gene specifically expressed by at least two specific types of inner ear cells. ProC: Transcription factor binding site (TFBS) repeats for multiple specific types of inner ear cell-specific transcription factors. ProD: Hypomethylated sequences of *cis*-acting elements of genes specifically expressed by specific types of inner ear cells.

The promoter sequences of specific expression genes are chosen using four different methods (Fig. 3). At the 5′ end of the gene specifically expressed in inner ear cells, the region between 500 and 3500 bp was selected to intercept the gene sequence as the synthesis promoter. The 5′ end sequence (synthetic promoter) is constructed using four different strategies [34]. ProA contains a sequence upstream of the initiation codon of a cell-specific gene in the inner ear of the mouse cochlea, the bases of the sequence at both ends of −1500 to 500 and −3000 to −1000 extracted from the 5′ −3000 to 500 bp of the gene specifically expressed in the inner ear cell. ProB is an ordered assembly of systemically inherited and conserved DNA elements identified in nucleotide sequences prior to at least two hair-cell-specific gene transcription initiation sites. The conserved genetic sites were predicted by the database of the University of California Santa Crus and National Center for Biotechnology. ProC is composed of multiple inner ear cell-specific repeat sequences of transcription factor binding sites (TFBS) and random sequence crossover. TFBS can be predicted by searching the literature and JASPER database [51]. ProD was determined based on the combination of epigenetics and transcriptome analysis. The hypomethylation sequence of *cis*-acting elements specifically expressed by inner ear cells could be predicted by MethPrimer and other databases. This part of hypomethylated *cis*-acting elements could be amplified from the genome as the synthesis promoter of ProD. ProC and ProD also contain the minimal TATA box synthetic promoter (minP) element. These studies are intended to obtain synthetic promoters of genes specifically expressed in inner ear cells, and prepare for the next step of in vivo screening.

By using these strategies to choose the promotor sequence of specific genes in cochlea cells, we are able to generate the highly transducing AAV variants in HCs, SCs, or SGNs (data not shown). It is a substantial amount of work to generate these AAV variants and screen for their transducing efficiency and specificity. We will continue these efforts to optimize AAV variants, which can transduce the cochlea cells in adult mice and other large animal models, such as pigs and nonhuman primates.

## Large animal models for hearing research

While most work related to inner ear gene therapy is conducted in rodents, larger animals, such as pigs or nonhuman primates, which have ears that are closer to those of humans, are better animal models for evaluating the efficiency/specificity and toxicity of AAV variants. Thus, these large animal models may extend translational proof-of-principle studies. China has established the largest pool of pig models and a large amount of mutations have been generated [52, 53]. Interestingly, the mutation of *SOX10* (R109W) in pigs by N-ethyl-N-nitrosourea mutagenesis causes inner ear malfunctions and hearing loss [52], and might represent a good model to test the gene therapy approach for hearing loss. Right now, we are conducting collaborative experiments to evaluate the efficiency/specificity and toxicity of AAV variants in pigs and expect some AAV variants may present as potential options to be used in clinical trials.

It is an exciting time for inner ear gene therapy. With the advent of new AAV variants displaying high efficiency and specificity in transducing cochlea cells and with the establishment of large animal models, such as pigs and nonhuman primates, we would expect the rapid translation from basic research to clinical trials is feasible. There are more than 100 different genes causing genetic hearing loss, yet the mechanisms underlying hearing dysfunction by distinct gene mutations are different and need to be fully investigated before developing the gene therapy strategy for each hearing deaf gene. Despite many challenges, there are reasons for optimism as new AAV variants, which specifically and efficiently target different cochlea cells, are developed and more collaborative projects, from both basic scientists and clinical doctors, are conducted to develop feasible gene therapy strategies for hearing loss.
